# Effects of oligo-fucoidan on the immune response, inflammatory status and pulmonary function in patients with asthma: a randomized, double-blind, placebo-controlled trial

**DOI:** 10.1038/s41598-022-21527-3

**Published:** 2022-10-28

**Authors:** Chia-wei Yeh, Chia-Ju Shih, Tu-Chen Liu, Ya-ling Chiou

**Affiliations:** 1 Master Program of Biomedical Nutrition Program, Department of Nutrition, Hungkuang University, No. 1018, Sec. 6, Taiwan Boulevard, Shalu District, Taichung, 43302 Taiwan, ROC; 2 Department of Chest Medicine, Cheng-Ching General Hospital, Taichung, Taiwan, ROC

**Keywords:** Asthma, Interleukins

## Abstract

Asthma is a common disease occurring worldwide. The clinical treatment of asthma is constantly revised and updated; however, it is associated with side effects. Our previous in vitro and ex vivo studies found that oligo-fucoidan can improve allergic immune responses and reduce airway inflammation. The purpose of this clinical trial was to investigate the effects of oligo-fucoidan on the immune status, inflammatory response, and pulmonary function of patients with asthma. Twenty asthmatic patients were randomly divided into two groups: (1) control group: receiving regular asthma treatment and supplementation with placebo; (2) fucoidan group: receiving regular asthma treatment and supplementation with oligo-fucoidan. Pulmonary function tests, the "Asthma Control Questionnaire" survey, biochemical data, inflammatory factors, and immune cell subtypes were detected. During treatment, the levels of WBC (p = 0.038) and creatinine (p = 0.012 and p = 0.008 at 12th and 24th weeks) were significantly decreased in the fucoidan group. Lung function (FEV_1_/FVC pr) significantly increased in the fucoidan group (p = 0.046). Regarding the proportion of immune cells, the level of IFN^+^ and CD4^+^IFN^+^cells in the fucoidan group was significantly increased during the treatment period (P < 0.05), while the proportions of CD3^+^CD4^+^ cells (p = 0.048) and CD3^+^CD8^+^ cells (p = 0.009) in the fucoidan group were significantly decreased during the treatment period. Regarding cytokines, the level of interleukin-8 (IL-8) was also significantly reduced in the fucoidan group during the treatment period.

## Introduction

Asthma is a chronic airway disease characterized by airway hyperresponsiveness, inflammation, and remodeling^1^. The global prevalence of asthma in adults varies widely from 1 to 18%^2^. The number of annual prevalent cases of diagnosed asthma increased from 56,885 in 2000 to 101,535 in 2011, a 2-fold increase over 11 years^3^. Asthma is characterized by chronic airway inflammation. Repeated airway inflammation causes epithelial cell injury, smooth muscle proliferation or hypotrophy, and secretory cell proliferation, resulting in increased mucous production^4^. This eventually results in airway hyperresponsiveness and remodeling, poor lung function, and difficulty in breathing.

In the pathogenesis of asthma, T cells play an important role in the regulation of chronic inflammatory responses in the respiratory tract. The upregulation of helper T type 1 (Th1) cells can reduce the inflammatory response in the respiratory tract induced by helper T type 2 (Th2) cells^5^. The cause of airway inflammation is complex and involves many cells and inflammatory factors. Consequently, drugs that control airway inflammation target several different pathways that produce inflammatory factors. Drug-based strategies to control airway inflammation include the use of corticosteroids or the combined use of an inhaled corticosteroid and a long-acting β2 agonist^6^ and seek alternative treatments. Cell and animal experiments related to nutritional adjunctive therapy, an alternative treatment, have shown that vitamins A, E, and C, folate, and fish oil can relieve asthma symptoms^7–11^. Fucoidan is a polysaccharide derived from brown seaweed extract. It is structurally similar to a heparin molecule; it consists of repeating units of disaccharides containing an alpha-1, 3-linked fructose and an alpha-1, 4-linked fructose, forming an alpha-1, 3-backbone with branches attached at C2 positions^12,13^. Fucoidan is reported to possess antiviral, antioxidant, antimicrobial, anticoagulant, anticancer/antitumor, antiproliferative, and anti-inflammatory properties^14–17^. Treatment of atopic allergic reactions with fucoidan extracted from various seaweeds could improve allergic responses by regulating immune responses, including alteration of the Th1/Th2 balance, inhibition of IgE production, and suppression of mast cell degranulation, and has the potential to prevent or reduce symptoms of allergic disease^18,19^. In our previous study, we found that oligo-fucoidan might reduce the proliferation of airway smooth muscle (ASM) cells^20^ and improve the balance of Th1/Th2 and Treg/Th17 ratios in asthmatic patients in ex vivo^21^. In this study, we evaluated the effects of oligo-fucoidan on immune status, inflammatory response, and pulmonary function in asthmatic patients in a clinical trial.

## Materials and methods

### Study subjects

**Figure 1:**
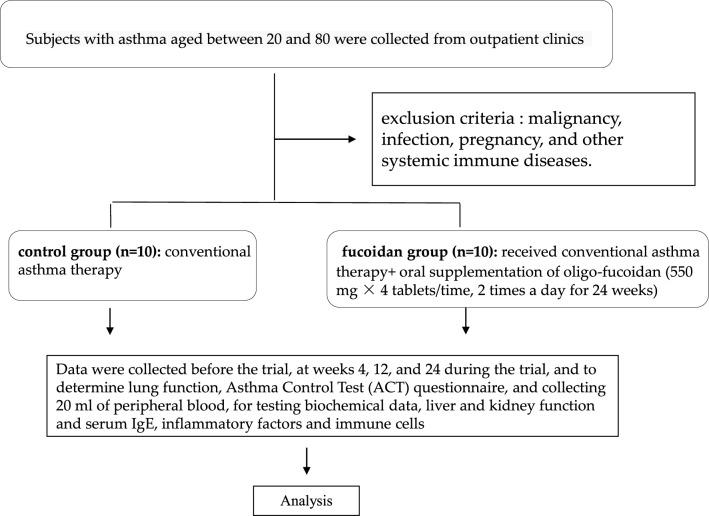
Flowchart for collecting subjects.

The subjects were outpatients at the Kuang-Tien General Hospital. The inclusion criteria were (1) presence of allergic asthma, (2) absence of complicated underlying disease, (3) lack of history of upper or lower airway disease during the month before the study, and (4) not having received immunotherapy or oral or intravenous steroids during the four weeks before the study (inhaled steroids were allowed). The exclusion criteria were malignancy, infection, pregnancy, and other systemic immune diseases. Twenty patients with allergic asthma were included in this study and Project Moderator and Research Assistant used block randomization divided into two groups: (1) control group (n = 10): conventional asthma therapy (2) fucoidan group (n = 10): received conventional asthma therapy oral supplementation of oligo-fucoidan (550 mg × 4 tablets/time, 2 times a day for 24 weeks) (Fig. 1). We use the medical record number and date as the registration mark, do not use the serial number. All subjects were fully aware of the purpose and nature of the study, which was approved by the Institutional Review Board (IRB) of Kuang-Tien General Hospital. This study was registered retrospectively, and registration number TCTR20220624008 and the date of registration was 24/06/2022.

### Asthma control test

The asthma control test (ACT) is a simple test suitable for asthma patients (aged 12 years and over) that helps determine the level of their control in the past 4 weeks. The test contained five questions; the lowest score was 1, and the highest score was 5 for each item; the lower the score, the worse the asthma control. The sum of the scores of the five questions, 25, was the maximum. A total score greater than 19 (excluding 19) indicated good asthma control, and a sum score of less than 19 indicated poor asthma control^22^..

### Lung function test

Pulmonary function was assessed using spirometry according to the standards of the American Thoracic Society. Asthma is diagnosed using spirometry, which includes measurement of FVC and FEV_1_. Bronchoprovocation (methacholine challenge test) is the stimulation of methacholine, which causes airway smooth muscle to contract, and the degree of bronchial stenosis is determined using the value of lung function, thereby confirming airway hyperresponsiveness (AHR).After inhalation of methacholine, if the measured value of lung function is significantly decreased (FEV_1_ decreased by more than 20%) or symptoms such as asthma are provoked, it may be considered as suspected asthma.

### Analysis of basic data and biochemical data of subjects

We collected anthropometric data, including body weight, height, and blood pressure, from the subjects using standardized techniques (IRB approved). Heparinized blood was collected to measure alanine aminotransferase (ALT), aspartate aminotransferase (AST), blood urea nitrogen (BUN), and creatinine (Cre). Total serum IgE levels were determined using a two-site sandwich immunoassay automated chemiluminescence system (Centaur XP Immunoassay System; Siemens Healthcare Diagnostics, Tarrytown, NY, USA). The remaining blood was separated into serum and cells. Serum was stored as aliquots in liquid nitrogen until analysis.

### Analysis of T cell subtypes

Half of the total cells were incubated with phycoerythrin (PE)-conjugated anti-human CD3 antibodies for 20 min at 4 °C in the dark and were separated and stained with fluorescein isothiocyanate (FITC)-conjugated anti-human CD4, anti-human CD8, or anti-human CD25 antibodies. The other half of the total cells were stained with FITC-conjugated anti-human CD4, resuspended in fixation and permeabilisation solution, and stained with PE-conjugated anti-human IFN-γ, anti-human IL-4, anti-human IL-17A, or anti-human Foxp3 antibodies. PE-conjugated mouse IgG1 antibodies were used as isotype controls. The antibodies were purchased from BD Biosciences (Franklin Lakes, NJ, USA).

### Analysis of the levels of cytokines

The serum were analyzed for cytokine levels, including IL-1β, IL-4, IL-6, IL-8 , IL-17α, IFNγ, using ELISA kits (eBioscience, San Diego, CA, USA). All samples were analyzed in duplicate.

### Statistical analysis

The results are expressed as the mean ± SD. Statistical Product and Service Solutions (SPSS) for Windows (SPSS Inc., Chicago, IL, USA) was used for statistical analyses. Statistical significance was determined by unpaired t-test for comparisons between asthma and non-asthma groups and by one-way ANOVA for comparisons between different treatment groups. Two-tailed statistical tests were used, and *p* < 0.05 indicated a statistically significant difference.

### Institutional review board statement

The study was conducted in accordance with the Declaration of Helsinki, and approved by the Institutional Review Board of Kuang-Tien General Hospital (10638, 31/01/2018). This study was registered retrospectively, and registration number TCTR20220624008 and the date of registration was 24/06/2022.

### Informed consent statement

Informed consent was obtained from all subjects involved in the study.

## Results

### Patient characteristics

Twenty asthmatic patients were recruited for the study and randomly divided into two groups: (1) control group (n = 10), receiving regular asthma treatment and supplementation with placebo; (2) fucoidan group (n = 10), receiving regular asthma treatment and supplementation with oligo-fucoidan (550 mg × 4 tablets/time, 2 times a day for 24 weeks). The basic demographic characteristics of the two groups are shown in Table 1. The WBC count in the fucoidan group decreased at the 4th week (6.3 ± 1.2) and the 24th week (5.9 ± 1.1) compared to that at baseline (7.4 ± 1.3). In addition, the level of cholesterol in the fucoidan group was increased in the 24th week (200.8 ± 20.5) compared with baseline (181.0 ± 19.7) (p < 0.05), but the level of cholesterol in the fucoidan group remained within the standard range.

**Table 1:** The characteristics of subjects.

	Control group (n = 10)				Fucoidan group (n = 10)			
	0 W	4 W	12 W	24 W	0 W	4 W	12 W	24 W
Age (y/o)	52.3 ± 18.8				55.0 ± 15.6			
BMI (kg/m^2^)	24.1 ± 4.7				23.4 ± 3.6			
ACT score	20.6 ± 3.4	18.7 ± 4.1	20.0 ± 4.2	19.5 ± 4.4	20.8 ± 4.1	21.7 ± 2.8	21.4 ± 2.6	21.7 ± 4.9
GOT (U/L)	25.5 ± 9.5	29.0 ± 12.5	27.0 ± 9.7	23.4 ± 7.0	22.7 ± 6.8	23.84 ± 3.8	25.2 ± 6.4	21.9 ± 4.2
GPT (U/L)	26.2 ± 10.5	28.7 ± 8.5	25.22 ± 4.98	25.0 ± 6.5	21.6 ± 7.5	23.40 ± 5.3	25.0 ± 9.8	24.8 ± 14.0
BUN (mg/dL)	14.8 ± 7.5	13.9 ± 3.6	14.9 ± 3.8	13.1 ± 3.8	12.3 ± 2.83	12.1 ± 4.0	13.1 ± 2.6	13.0 ± 3.5
Creatinine (mg/dL)	0.9 ± 0.2	0.9 ± 0.3	0.9 ± 0.2	0.9 ± 0.2	0.9 ± 0.4	0.9 ± 0.2	0.9 ± 0.3	0.8 ± 0.3
TG (mg/dL)	138.7 ± 83.3	156.8 ± 79.5	135.2 ± 47.5	139.1 ± 77.7	141.3 ± 61.3	171.1 ± 83.9	176.8 ± 145.7	174.9 ± 90.5
Choloesterol(mg/dL)	196.7 ± 44.7	196.3 ± 23.1	205.6 ± 21.2	215.0 ± 18.2	181.0 ± 19.7	182.1 ± 21.5	182.1 ± 20.8	200.8 ± 20.5*
WBC (103/μL)	8.2 ± 2.0	7.7 ± 1.9	8.0 ± 2.5	8.3 ± 2.5	7.4 ± 1.3	6.3 ± 1.2*	6.4 ± 1.44	5.9 ± 1.1*
Lymphocyte (%)	26.3 ± 6.9	27.4 ± 7.3	26.8 ± 6.9	28.1 ± 7.4	30.5 ± 4.7	33.3 ± 5.5	34 ± 5.9	32.3 ± 6.7
Neutrophil (%)	63.6 ± 8.5	61.5 ± 9.4	63.6 ± 7.8	61.9 ± 9.9	60.7 ± 6.5	56 ± 7.2	54.8 ± 8.4	58.3 ± 8.8
Monocyte (%)	5.8 ± 1.7	6.5 ± 2.3	6.1 ± 2.9	5.8 ± 2.1	5.7 ± 0.9	5.7 ± 0.8	5.6 ± 1.5	5.3 ± 0.8
Eosinophil (%)	3.6 ± 3.6	4.0 ± 2.4	2.9 ± 2.3	3.4 ± 1.9	3.2 ± 1.5	4.1 ± 1.0	4.4 ± 1.7	3.5 ± 2.0
Basophil (%)	0.8 ± 0.6	0.8 ± 0.4	0.5 ± 0.5	0.3 ± 0.5	0.5 ± 0.5	0.7 ± 0.8	0.5 ± 0.5	0.8 ± 0.4
Total IgE (kUA/L)	283.51 ± 681.3	351.5 ± 883.2	381.0 ± 945.7	365.7 ± 875.6	327.6 ± 355.1	205.5 ± 225.3	289.0 ± 347.5	257.9 ± 239.4
FVC (L)	3.4 ± 0.9	3.4 ± 0.9	3.5 ± 0.9	3.4 ± 0.9	3.5 ± 0.8	3.4 ± 0.9	3.5 ± 0.8	3.4 ± 0.8
FEV1(L)	2.8 ± 0.8	2.8 ± 0.8	2.9 ± 0.8	2.8 ± 0.8	2.9 ± 0.7	2.8 ± 0.7	2.8 ± 0.7	2.8 ± 0.7
FEF (25–75%)	3.0 ± 0.9	3.0 ± 0.9	3.1 ± 0.9	3.0 ± 1.0	3.0 ± 0.8	2.9 ± 0.8	3.0 ± 0.8	2.9 ± 0.8
FEV1/FVC	81.3 ± 2.6	81.3 ± 2.6	81.5 ± 2.5	81.2 ± 2.6	81.0 ± 2.0	81.5 ± 1.6	81.0 ± 2.0	80.6 ± 2.3

*The data during 4th, 12th, 24th week (4 W, 12 W, 24 W) compared with baseline (0 W) had significant different (p < 0.05).

### The changes in biochemical data of patients before and after supplementation with oligo-fucoidan

Changes in the biochemical data of patients before and after supplementation with oligo-fucoidan are listed in Table 2. The changes in creatinine (Cre) at the 12th week (−0.1 ± 0.3) and 24th week (−0.2 ± 0.4) during oligo-fucoidan supplementation decreased more significantly than those in the control group at the 12th week (0.03 ± 0.07) and 24th week (0.07 ± 0.06) (p = 0.012 and p = 0.008). The change in WBC at 24th week (−1482.2 ± 1285.7) during supplementation with oligo-fucoidan decreased significantly more than that in the control group at 24th week (−589.0 ± 2835.0) (p = 0.038). With respect to lung function, the change in FEV1/FVC at the 4th week (0.5 ± 1.1) during supplementation with oligo-fucoidan increased significantly compared to the that of the control group (0.1 ± 0.11) at the 4th week (p = 0.046). In addition, we also found that the levels of total IgE in the 12th week (38.6 ± 125.6) and 24th week (−69.7 ± 139.1) during supplementation with oligo-fucoidan decreased compared to that in the control group at the 12th week (97.5 ± 264.6) and 24th week (82.2 ± 196.4), but the difference was not statistically significant.

**Table 2:** The changed of biochemical data and lung function during supplementation period.

	Control group (n = 10)			Fucoidan group (n = 10)		
	△4 W	△12 W	△24 W	△4 W	△12 W	△24 W
GOT (U/L)	3.4 ± 4.9	1.3 ± 1.7	−2.2 ± 5.0	1.1 ± 5.9	2.5 ± 4.0	−0.9 ± 4.6
GPT (U/L)	2.7 ± 9.8	−0.8 ± 10.0	−0.7 ± 7.3	1.8 ± 5.61	3.4 ± 7.3	2.7 ± 12.3
BUN (mg/dL)	−0.9 ± 5.7	0.1 ± 6.11	−1.7 ± 7.5	−4.2 ± 14.3	0.8 ± 2.7	0.7 ± 3.1
Creatinine (mg/dL)	0.1 ± 0.2	0.03 ± 0.07	0.07 ± 0.06	−0.1 ± 0.2	−0.1 ± 0.3*	−0.2 ± 0.4*
TG (mg/dL)	16.5 ± 54.3	−3.2 ± 82.9	0.4 ± 56.8	29.7 ± 54.1	32.5 ± 134.8	33.6 ± 84.8
Cholesterol (mg/dL)	−0.3 ± 38.7	8.9 ± 29.9	18.2 ± 38.3	1.2 ± 22.4	1.1 ± 22.5	19.7 ± 22.6
WBC (10^3^/μL)	−474.4 ± 1574.1	−161.0 ± 1636.7	−589.0 ± 2835.0	−1194.4 ± 1078.6	−1087.0 ± 1691.3	−1482.2 ± 1285.7*
Lymphocyte (%)	1.25 ± 6.1	0.33 ± 4.7	1.89 ± 8.46	−0.2 ± 2.8	2.5 ± 6.9	2.67 ± 7.3
Neutrophil (%)	−1.25 ± 8.1	0.56 ± 6.8	−1.11 ± 11.96	−0.8 ± 2.5	−4.83 ± 10.2	−3.67 ± 10
Monocyte (%)	0.63 ± 1.3	0.67 ± 1.73	0 ± 1.41	0.4 ± 0.5	1 ± 1.67	0.17 ± 0.4
Eosinophil(%)	0.3 ± 2.0	−0.7 ± 2.3	−0.2 ± 3.8	0.9 ± 1.3	1.2 ± 2.1	0.4 ± 2.4
Basophil (%)	−1.3 ± 0.3		−0.33 ± 0.7		0.2 ± 0.45	
Total IgE (kUA/L)	−209.1 ± 655.5	97.5 ± 264.6	82.2 ± 196.4	−123.6 ± 277.4	−38.6 ± 125.6	−69.7 ± 139.1
FVC (L)	−0.003 ± 0.009	0.04 ± 0.2	−0.01 ± 0.01	−0.3 ± 0.5	−0.01 ± 0.01	−0.05 ± 0.08
FEV1 (L)	−0.03 ± 0.01	0.04 ± 0.13	−0.014 ± 0.015	−0.2 ± 0.4	−0.01 ± 0.01	−0.2 ± 0.3
FEF (25–75%)	−0.003 ± 0.009	−0.06 ± 0.16	−0.015 ± 0.019	−0.2 ± 0.3	−0.01 ± 0.02	−0.2 ± 0.4
FEV1/FVC	0.1 ± 0.1	0.2 ± 0.6	−0.11 ± 0.13	0.5 ± 1.1*	−0.01 ± 0.12	−0.4 ± 1.2

△: Indicates the data of week 4 or 12 or 24 minus the data of week 0.

*: Indicates the data subtracted from the 4th week or the 12thweek or the 24thweek and the 0thweek, there is a significant difference between the control group and the fucoidan group (p < 0.05).

### The increased proportion of IFN^+^ cells in asthmatic patients after supplementation with fucoidan

The proportion of immune cells before and after fucoidan supplementation is shown in Table 3. The surface antigen markers of immune cells were measured including CD3CD4, CD3CD8, CD4CD25, IFN, CD4IFN, CD4IL-4, CD4IL-17, CD4FoxP3 by flow cytometry at baseline, 4th, 12th, and 24th week of supplementation with fucoidan. The results showed that the proportion of IFN^+^ and CD4^+^IFN^+^ cells in the 24th week (55.5 ± 13.99,17.9 ± 8.1) was significantly higher than that in the 0th week (baseline) (26.3 ± 24.1, 10.2 ± 11.3) (P < 0.05) in the fucoidan group, and there was no significant difference in other subsets of immune cells.

**Table 3:** The proportion of immune cell types in the two groups during supplementation period.

	Control group (n = 10)				Fucoidan group (n = 10)			
	0 W	4 W	12 W	24 W	0 W	4 W	12 W	24 W
CD3CD4 (%)	36.8 ± 3.8	34.4 ± 7.5	36.7 ± 4.9	34.9 ± 8.6	45.3 ± 12.0	42.6 ± 12.8	43.8 ± 12.3	40.3 ± 12.4
CD3CD8 (%)	20.3 ± 5.8	23.1 ± 5.8	22.5 ± 6.4	21.4 ± 6.1	22.9 ± 8.2	24.0 ± 8.6	21.0 ± 7.0	22.9 ± 7.5
CD4CD25 (%)	3.5 ± 0.7	3.6 ± 1.7	3.3 ± 0.5	2.9 ± 1.4	3.2 ± 0.2	3.3 ± 1.5	2.9 ± 1.1	2.4 ± 0.3
IFN (%)	30.9 ± 29.2	27.9 ± 15.6	32.7 ± 17.8	36.2 ± 18.8	26.3 ± 24.1	37.4 ± 25.1	40.2 ± 17.3	55.5 ± 14.0*
CD4IFN (%)	11.1 ± 10.6	9.3 ± 4.7	11.9 ± 7.9	11.6 ± 6.2	10.2 ± 11.3	13.7 ± 7.5	14.8 ± 10.0	17.9 ± 8.1*
CD4IL-4 (%)	0.01 ± 0.03	0.04 ± 0.07	0	0.01 ± 0.03	0.01 ± 0.03	0.02 ± 0.04	0.01 ± 0.03	0.01 ± 0.03
CD4IL-17 (%)	0.03 ± 0.05	0.04 ± 0.05	0.02 ± 0.04	0.04 ± 0.05	0.03 ± 0.05	0	0.03 ± 0.05	0.02 ± 0.06
CD4FoxP3 (%)	0.07 ± 0.08	0.1 ± 0.2	0.02 ± 0.04	0.04 ± 0.05	0.03 ± 0.05	0.03 ± 0.07	0.05 ± 0.07	0.03 ± 0.05

*: Indicates that there is a significant difference between the 4thweek, the 12thweek, the 24thweek and the 0thweek of the fucoidan group (*p* < 0.05).

**Table 4:** The changed proportion of immune cell types in the two groups during supplementation period.

	Control group (n = 10)			Fucoidan group (n = 10)		
	△4 W	△12 W	△24 W	△4 W	△12 W	△24 W
CD3CD4 (%)	−2.4 ± 7.6	−0.1 ± 3.9	−1.9 ± 7.0	−2.7 ± 8.9	−1.4 ± 8.6*	−4.9 ± 8.8
CD3CD8 (%)	2.8 ± 7.1	0.6 ± 10.3	1.0 ± 6.3	1.1 ± 3.0*	−1.9 ± 4.5	−3.4 ± 6.7
CD4CD25 (%)	0.2 ± 2.2	−0.2 ± 0.8	−0.5 ± 1.2	0.02 ± 0.96	−0.3 ± 1.4	−0.8 ± 1.4
IFN (%)	−3.0 ± 32.0	1.8 ± 38.9	2.8 ± 37.9	11.1 ± 22.9	10.4 ± 36.8	39.6 ± 13.4*
CD4IFN (%)	−3.0 ± 11.2	0.8 ± 15.7	0.5 ± 12.6	4.7 ± 10.0*	6.4 ± 15.8*	7.7 ± 14.9*
CD4IL-4 (%)	0.03 ± 0.08	−0.01 ± 0.03	0 ± 0.1	0.01 ± 0.06	0 ± 0.05	0 ± 0.05
CD4IL-17 (%)	0.01 ± 0.07	0 ± 0.07	0.01 ± 0.03	−0.03 ± 0.05	0 ± 0.07	−0.01 ± 0.09
CD4FoxP3 (%)	0.05 ± 0.21	−0.05 ± 0.08	−0.03 ± 0.10	0 ± 0.08	0.02 ± 0.08	0 ± 0.05

△: Indicates the data of week 4 or 12 or 24 minus the data of week 0.

*: Indicates the data subtracted from the 4th week or the 12th week or the 24th week and the 0th week, there is a significant difference between the control group and the fucoidan group (p < 0.05).

### The changed proportions of immune cells in asthmatic patients before and after supplementation with fucoidan

The results comparing the changes in the proportion of immune cells in the two groups of subjects during the treatment period are shown in Table 4. The results showed that the proportion of CD3^+^CD4^+^ cells was significantly decreased in the fucoidan group at the 12th week (−1.4 ± 8.6) compared with the control group (−0.1 ± 3.9) (p = 0.048). In addition, the proportion of CD3^+^CD8^+^ cells were significantly decreased in the fucoidan group in the 4th week (1.1 ± 3.0) compared with the control group (−2.8 ± 7.1) (p = 0.009). However, the proportion of IFN ^+^ and CD4^+^IFN^+^ cells are more significantly increased in the Fucoidan group in the 4th week (11.1 ± 22.9, 4.7 ± 10.0), 12th week (10.4 ± 36.8,6.4 ± 15.8), and 24th week (39.6 ± 13.4, 7.7 ± 14.9) compared with the control group (p < 0.05).

### The decreased level of IL-8 in asthmatic patients after supplementation with fucoidan

**Figure 2:**
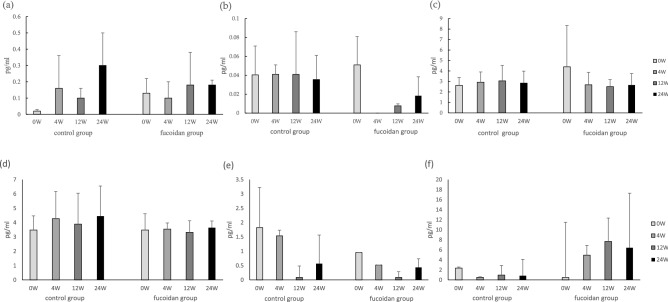
The concentrations of cytokines (**a**) IL-1β , (**b**) IL-4, (**c**) IL-6,(**d**) IL-8, (**e**) IL-17α, (**f**) IFNγ in the two groups during supplementation period.

**Figure 3:**
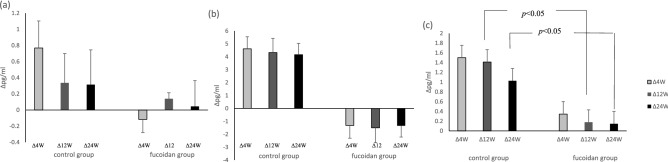
The changed concentrations of cytokines (**a**) IL-1β, (**b**) IL-6, (**c**) IL-8, in the two groups during supplementation period.

The levels of cytokines, including IL-1β, IL-4, IL-8, IL-6, IL-17, and IFN-γ, were analyzed using ELISA (Fig. 2). There were no statistically significant differences in the cytokine levels between the two groups during the treatment period. Changes in the levels of cytokines in the two groups of subjects during the treatment period are shown in Fig. 3. The results showed that the levels of IL-1β and IL-6 trend decreased in the fucoidan group at the 24th week compared with the control group; in particular, the levels of IL-8 significantly decreased in the fucoidan group at the 12th and 24th week compared with the control group (p < 0.05).

## Discussion

Previous studies have indicated that fucoidan prevents kidney damage by reducing inflammation, oxidative stress, and apoptosis^23–25^. In this study (Table 2), the levels of creatinine were significantly decreased in the fucoidan group at the 12th week (−0.092 ± 0.289) and 24th week (−0.157 ± 0.394) compared with the control group (0.032 ± 0.073, 0.068 ± 0.061) (p = 0.012 and p = 0.008), which was consistent with the results of previous studies. Oligo-fucoidan has been linked to the prevention of kidney damage and insufficiency.

Previous studies have indicated that oligo-fucoidan modulates immune responses, including altering the Th1/Th2 balance, inhibiting IgE production, inhibiting mast cell degranulation, and inhibiting ASM proliferation^20^, and IFN-γ has an inhibitory effect on airway inflammation produced by Th2 cells and can promote the regulation of Th1/Th2 balance. These results suggested that IFN-γ acts on airway epithelial cells to inhibit airway inflammation in allergic airway diseases^21^. Fucoidan may act like β-glucan on one or more membrane receptors on immune cells (including macrophages and dendritic cells) to stimulate activated T cells to differentiate toward Th1 and produce IFN-γ or differentiate into Treg cells and produce IL-10. Fucoidan may stimulate the activation of macrophages and dendritic cells by participating in scavenger receptor-A (SR-A), which can enhance antigen presentation and antigen specificity, and activate CD4^+^ and CD8^+^ T cells^26^, especially upregulate the proportion of IFN-γ-producing CD4^+^ and CD8^+^ T cells. The results of this study showed that oligo-fucoidan increased the proportion of IFN-γ-producing cells, especially CD4^+^IFN^+^ cells (Table 3), and the change in the Th1/Th2 balance is consistent with previous research results. Improving airway inflammation and airway hyperresponsiveness can increase pulmonary function (FEV_1_/FVC) (Table 1), suggesting its potential as adjuvant therapy for allergic asthma.

An imbalance between pro-inflammatory and anti-inflammatory factors in the pathogenesis of asthma leads to airway remodeling, which is related to the severity and progression of chronic lung inflammation. Studies on asthma indicate that inhibiting the production of inflammatory factors, such as IL-1, IL-6, IL-8, IL-4, IL-5, TNF-α, and IFN-γ, can reduce allergic symptoms^27,28^. In this study, the results indicated the proportion of CD3^+^CD4^+^ cells, CD3^+^CD8^+^ cells (Table 4), and the levels of inflammatory factors (Figs. 2 and 3) in the fucoidan group were significantly lower than those in the control group (p = 0.048).

Interleukin-8 (IL-8) is secreted by a variety of cell types, including monocytes, lymphocytes, endothelial cells, and bronchial epithelial cells. IL-8 is an inflammatory chemokine, causing the action of neutrophil chemoattractant^29^. In this study, IL-8 levels decreased significantly (p < 0.05) in the fucoidan group during the supplementation period (Fig. 3). A possible mechanism may be that oligo-fucoidan inhibits the number of lymphocytes to reduce the production of IL-8. In conclusion, oligo-fucoidan could reduce the proportion of lymphocytes and the concentration of inflammatory factors in patients with asthma to inhibit inflammation of the respiratory tract and increase pulmonary function. This study had a limitation that the number of subjects was small, and most of them were well-controlled asthma patients. Thus, the statistical comparison of the data showed no significant difference.

## Conclusions

Oligo-fucoidan could reduce the proportion of lymphocytes and the concentration of inflammatory factors in patients with asthma to inhibit inflammation of the respiratory tract and increase pulmonary function.

## Data Availability

The data that support the findings of this study are available from the corresponding author, upon reasonable request.
